# Improved In Vitro Test Procedure for Full Assessment of the Cytocompatibility of Degradable Magnesium Based on ISO 10993-5/-12

**DOI:** 10.3390/ijms20020255

**Published:** 2019-01-10

**Authors:** Ole Jung, Ralf Smeets, Philip Hartjen, Reinhard Schnettler, Frank Feyerabend, Martin Klein, Nils Wegner, Frank Walther, Dominic Stangier, Anders Henningsen, Carsten Rendenbach, Max Heiland, Mike Barbeck, Alexander Kopp

**Affiliations:** 1 Department of Oral Maxillofacial Surgery, Division of Regenerative Orofacial Medicine, University Medical Center Hamburg-Eppendorf, 20246 Hamburg, Germany; r.smeets@uke.de (R.S.); p.hartjen@uke.de (P.H.); reiner.schnettler@mac.com (R.S.); henningsen@gmx.de (A.H.); m.barbeck@uke.de (M.B.); 2 Department of Oral Maxillofacial Surgery, University Medical Center Hamburg-Eppendorf; 20246 Hamburg, Germany; 3 Institute of Materials Research, Division Metallic Biomaterials, Helmholtz-Zentrum Geesthacht, 21502 Geesthacht, Germany; frank.feyerabend@hzg.de; 4 Department of Materials Test Engineering (WPT), TU Dortmund University, 44227 Dortmund, Germany; martin.klein@tu-dortmund.de (M.K.); nils.wegner@tu-dortmund.de (N.W.); frank.walther@tu-dortmund.de (F.W.); 5 Institute of Materials Engineering, TU Dortmund University, 44227 Dortmund, Germany; dominic.stangier@tu-dortmund.de; 6 Charité-Universitätsmedizin Berlin, Corporate Member of Freie Universität Berlin, Humboldt-Universität zu Berlin, and Berlin Institute of Health, Department of Oral and Maxillofacial Surgery, 12200 Berlin, Germany; carsten.rendenbach@charite.de (C.R.); max.heiland@charite.de (M.H.); 7 Berlin Institute of Health, 10178 Berlin, Germany; 8 BerlinAnalytix GmbH, 12109 Berlin, Germany; 9 Meotec GmbH & Co. KG, 52068 Aachen, Germany; alexander.kopp@meotec.eu

**Keywords:** magnesium, ISO10993-5/-12, PEO, degradation, biocompatibility, implant

## Abstract

Magnesium (Mg)-based biomaterials are promising candidates for bone and tissue regeneration. Alloying and surface modifications provide effective strategies for optimizing and tailoring their degradation kinetics. Nevertheless, biocompatibility analyses of Mg-based materials are challenging due to its special degradation mechanism with continuous hydrogen release. In this context, the hydrogen release and the related (micro-) milieu conditions pretend to strictly follow in vitro standards based on ISO 10993-5/-12. Thus, special adaptions for the testing of Mg materials are necessary, which have been described in a previous study from our group. Based on these adaptions, further developments of a test procedure allowing rapid and effective in vitro cytocompatibility analyses of Mg-based materials based on ISO 10993-5/-12 are necessary. The following study introduces a new two-step test scheme for rapid and effective testing of Mg. Specimens with different surface characteristics were produced by means of plasma electrolytic oxidation (PEO) using silicate-based and phosphate-based electrolytes. The test samples were evaluated for corrosion behavior, cytocompatibility and their mechanical and osteogenic properties. Thereby, two PEO ceramics could be identified for further in vivo evaluations.

## 1. Introduction

Magnesium (Mg) is an essential element for different biochemical mechanisms in the human body including wound healing and the regeneration of soft and hard tissue [1,2,3]. For example, it acts as an important co-factor for many enzymes that are involved in protein and collagen synthesis [1,2,3]. Furthermore, it has been shown that Mg is involved in different direct and indirect processes that trigger bone growth such as the increased expression of collagen type X and the vascular endothelial growth factor (VEGF) of osteogenic cells [4]. Interestingly, Mg has also shown to provide mechanical strength similar to the human cortical bone, making it an ideal candidate for different medical devices such as bone substitutes, bone regeneration scaffolds, and osteosyntheses plates and screws [4,5,6,7]. Due to its natural biodegradability, the use of Mg-based materials can render secondary operations for removing non-degradable materials such as titanium plates or screws for bone fixation [4,8]. However, its degradation process leads to the release of hydrogen (H_2_) and the alkalization of the micro-environment, which can counteract proper healing of hard and soft tissues [9,10,11].

Thus, the degradation process of Mg-based implants has shown to limit their biocompatibility [12,13,14]. The successful development of magnesium materials for medical applications can be achieved through two strategies to ensure a controlled release of degradation products that correlates with the physiological regulation mechanisms: (a) the fabrication of corrosion-resistant magnesium alloys that decrease Mg corrosion and (b) the application of different surface coatings that delay the corrosion process up to its own degradation [6,15,16,17,18].

Plasma electrolytic oxidation (PEO), also known as micro-arc oxidation (MAO), is a promising method for surface modification of Mg implants [12,19]. In this process, Mg or Mg-alloys are immersed in an electrolytic bath where high-voltage currents create intense plasma on the metal surface with subsequent cooling and oxidization of the surface, yielding a micro-structured ceramic coating [12,20,21]. By controlling parameters such as the electrolyte composition, voltage, and time, surfaces of various thickness, roughness, and porosity can be achieved [12,20,21]. Thereby, the electrolytic composition plays an important role to achieve sufficient biocompatibility and surface activity [12,20,22]. In summary, PEO-treatment allows to improve the biocompatibility and mechanical properties of magnesium-based biomaterials to make them more suitable for applications even at load-bearing sites [21].

In this context, the development process of new Mg-based biomaterials has to include an adequate in vitro cytocompatibility testing in accordance with ISO 10993-5/-12 standard regulations as a partial basis for product registration [23]. The dissolution of magnesium under H_2_-release as well as the release of other degradation products have to be addressed, as these factors have a decisive impact on the overall biocompatibility [12,13]. Thereby, magnesium degradation is influenced by various conditions and media-components such as pH and concentrations of HCO_3_^−^, albumin, etc. in such a test setup, which can, along with the magnesium degradation products, interfere with the commonly used in vitro cytotoxicity analysis protocols [12,24,25,26,27,28,29,30]. Although different protocols including advanced test conditions for direct and extract-based tests have already been described considering the special degradation characteristics of Mg and its release products, the previously published test protocol developed by our group is more closely in accordance with ISO 10993-5/-12 [12,31,32,33,34,35]. This test scheme includes: (1) an indirect assessment of effects of soluble Mg corrosion products in material extracts and (2) a direct assessment of the surface compatibility in terms of cell attachment and cytotoxicity originating from active corrosion processes. The indirect assessment allows the quantification of cell proliferation, viability as well as cytotoxicity of mouse fibroblasts incubated with material extracts. Furthermore, the direct assessment visualizes cells attached to the test materials by means of live-dead staining. This experimental setup can be used for all Mg-based biomaterials. However, important aspects that can affect the in vivo-performance, such as mechanical tests or tests with osteogenic cells, should be considered.

Thus, the aim of the present study was the extension of the general in vitro-test protocol to include the characterization of mechanical and osteogenic material properties to optimize the development of magnesium materials and surfaces for bone tissue repair in terms of time and quality. Finally, this newly developed test protocol should allow more efficient in vivo trials and the prevention of extensive animal testing. The extended protocol was developed on basis of Mg test samples, which were ceramized by PEO. In a first step, 12 different Mg surfaces were tested for cytocompatibility and corrosion [23]. Only materials that were found suitable in this first step were further characterized focusing on suitable mechanical and osteogenic aspects. Both of these test steps were established in the present study as a new standardized test protocol for Mg-based biomaterials to optimize time, quality, and safety of Mg in in vivo-setups.

## 2. Results

### 2.1. Physical Features of WE43-PEO Variants

**Figure 1:**
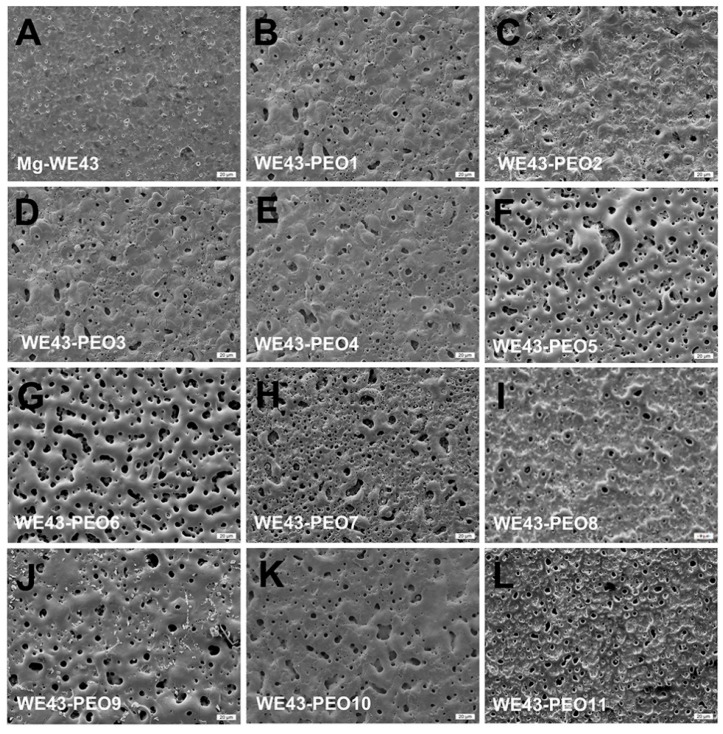
SEM pictures of WE43 (**A**), four silicate based ceramized-variants of WE43 (**B**–**E**) and six phosphate based PEO test samples (**F**–**L**). White bar is 20 μm.

Using different variations of PEO coatings, i.e., four silicate-based and seven phosphate-based coatings, the surface structures and roughnesses varied largely (Figure 1). While the uncoated WE43 alloy exhibited the smoothest surface with the lowest porosity without any micro-pores (Figure 1A), the silicate-coated WE43 variants exhibited a rougher surface structure with comparable moderate numbers of micropores and small diameters micropores (Figure 1B–E). In contrast, the phosphate-coated WE43 variants WE43-PEO5 and WE43-PEO6 showed a more textured surface pattern with bigger micropores and grooved structures (Figure 1F,G). WE43-PEO7 showed a textured surface but without high numbers of micropores (Figure 1H). Interestingly, the variant WE43-PEO8 showed a relatively smooth surface structure combined with moderate micropore numbers comparable to the silicate-coated WE43 variants (Figure 1I). WE43-PEO9 and WE43-PEO10 showed again highly textured surface structures comparably to WE43-PEO7 (Figure 1J,K). Finally, the variant WE-PEO11 exhibited a moderately textured surface but higher numbers of micropores (Figure 1L).

### 2.2. First Material Analyses

#### 2.2.1. Corrosion Measurements

**Figure 2:**
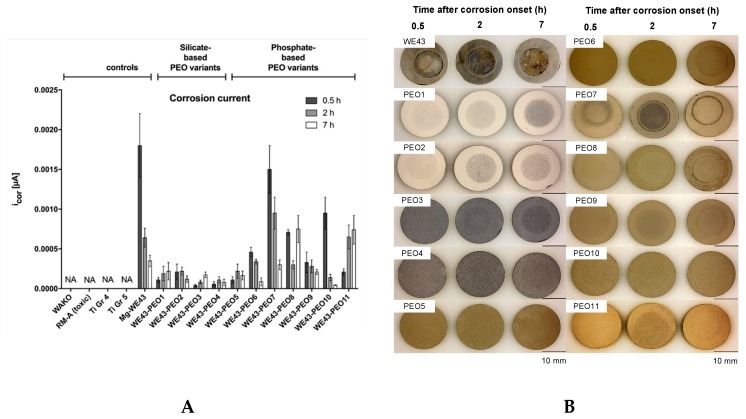
(**A**) Corrosion currents of the variants after 0.5, 2, and 7 h after placing the variants into minimum essential medium (MEM). (**B**) Appearance of the variants after 0.5, 2, and 7 h corrosion. NA: not applicable.

In total, the different corrosion patterns could be assessing for the time points 0.5, 2, and 7 h: (1) increasing, (2) decreasing, and (3) mixed. All silicate-based test samples showed increasing or mixed patterns on a relatively low level. Mg-WE43, WE43-PEO6, WE43-PEO7, WE43-PEO9, and WE43-PEO 10 showed decreasing patterns. WE43-PEO5 and WE43-PEO8 revealed mixed values, while WE43-PEO11 showed increasing rates. Mg-WE43 and WE43-PEP7 exhibited the highest initial corrosion 0.5 h after being placed in the medium, followed by PEO7 and PEO10. All other PEO variants exhibited very low initial corrosion current of below 0.001 μA (Figure 2A,B). For WE43, PEO6, PEO7, PEO9, and PEO10, the initial corrosion current was higher than the other two corrosion currents 2 and 7 h later. By contrast, in the case of WE43-PEO8 and WE43-PEO11, the last corrosion current at 7 h was the highest (Figure 2A,B).

#### 2.2.2. pH Measurements

**Figure 3:**
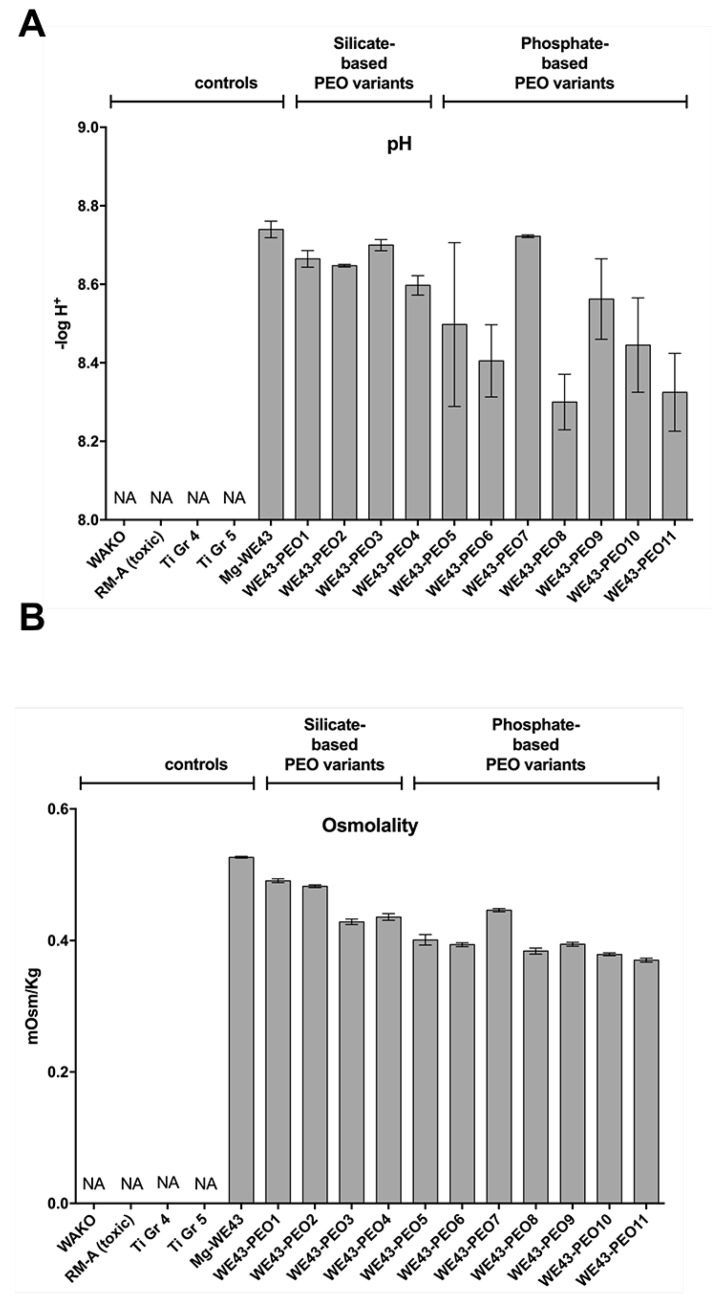
Chemical features of PEO variants. pH (**A**) and Osmolality (**B**) were measured in medium in which the variants were placed for 3 days. Column heights are means with error bars indicating standard deviations. NA: not applicable.

When placed in cell culture medium, corrosion of untreated WE43 led to a pH of 8.74 ± 0.02 (Figure 3A). Interestingly, the pH associated with the silicate-modified variants showed comparable values (WE43-PEO1: 8.67 ± 0.02, WE43-PEO2: 8.65 ± 0.02, WE43-PEO3: 8.70 ± 0.01, and WE43-PEO4: 8.59 ± 0.02) without significant differences between the values of the silicate-based materials (Figure 3A).

Most of the phosphate-coated materials showed significantly lower pH values compared to both the uncoated WE43 material and the silicate-coated study groups with exception of the pH values in the WE43-PEO7 (8.72 ± 0.03) and the WE43-PEO9 group (8.56 ± 0.07) (Figure 3A). The values in case of the uncoated Mg materials were significantly increased compared to the values in the WE43-PEO5 group (8.48 ± 0.15, *** *p* < 0.001), the WE43-PEO6 group (8.39 ± 0.07, *** *p* < 0.001), WE43-PEO8 (8.3 ± 0.05, *** *p* < 0.001), WE43-PEO9 (*** *p* < 0.05), WE43-PEO10 (8.45 ± 0.09, *** *p* < 0.001), and WE43-PEO11 (8.33 ± 0.07, *** *p* < 0.001) (Figure 3A). Furthermore, significantly lower pH values were found in the WE43-PEO6 group (*** *p* < 0.001), the WE43-PEO8 group (*** *p* < 0.001) and the WE43-PEO11 group (*** *p* < 0.001) (Figure 3A). Interestingly, no significant differences were found comparing the values of the WE43-PEO2 group and the WE43-PEO5 group, the values in WE43-PEO4 group and the WE43-PEO5 group as well as in the WE43-PEO4 group and the WE43-PEO10 group (Figure 3A). Moreover, significant differences were found comparing the values in the WE43-PEO1 and the WE43-PEO3 groups with the WE43-PEO2 group (* *p* < 0.05 and *** *p* < 0.001) (Figure 3A). Also, significant differences were calculated when comparing the values in the groups WE43-PEO1, WE43-PEO2, and WE43-PEO3 with that found in the WE43-PEO10 group (** *p* < 0.01) (Figure 3A).

The statistical comparison of the values in the phosphate-based material groups additionally showed that the values in the WE43-PEO5 group only differed significantly compared to that detected in the WE43-PEO7 group and the WE43-PEO8 group (*** *p* < 0.001 and * *p* < 0.05) (Figure 3A). The values in the WE43-PEO6 group only differed significantly compared to the values in the WE43-PEO7 group (*** *p* < 0.001) (Figure 3A). Moreover, the values in the WE43-PEO7 group differed significantly compared with that found in the WE43-PEO8 group, the WE-PEO10 group and the WE43-PEO11 group (*** *p* < 0.001 and * *p* < 0.05) (Figure 3A). Additionally, significant differences were calculated between the values in the WE43-PEO8 group and that found in the WE43-PEO9 group (*** *p* < 0.001), while no differences were detected between the values in the WE43-PEO9 group, the WE43-PEO10 group and the WE43-PEO11 group (Figure 3A).

Finally, all pH values in the groups of the Mg-based materials were significantly higher compared with the values in the control groups, i.e., the WAKO group, the RM-A group and both titanium groups that did not show any pH values (*** *p* < 0.001) (Figure 3A).

#### 2.2.3. Osmolality Measurements

**Figure 4:**
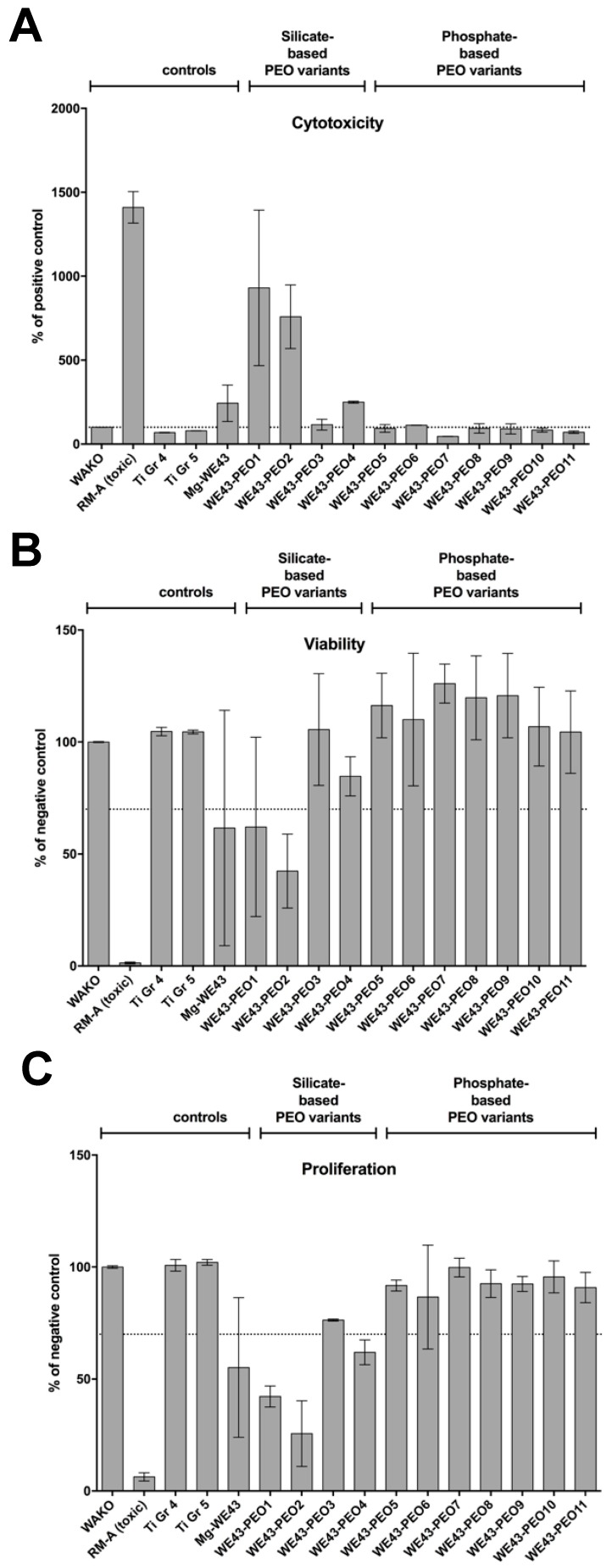
Cytocompatibility of the plasma electrolytic oxidation (PEO) variants. (**A**) Cytotoxicity measured by a Lactate Dehydrogenase (LDH) assay; (**B**) viability measured by a Sodium 3,3′-[1(phenylamino)carbonyl]-3,4-tetrazolium]-3is(4-methoxy-6-nitro) Benzene Sulfonic acid Hydrate (XTT)-assay; (**C**) proliferation measured by a BrdU assay. Values are either normalized against positive controls (LDH) or negative control (XTT, BrdU). Means with error bars indicating standard deviations. The dotted line indicates thresholds which should not be exceeded (LDH) or fall below (XTT; BrdU).

The measurements of the osmolality of the Mg alloy and the differently coated materials showed that the values in the WE43 group (0.53 ± 0.0006 mOsm/Kg) were significantly higher compared to all coated Mg-based materials, i.e., WE43-PEO1 to WE43-PEO11 (*** *p* < 0.001) (Figure 3B). Moreover, also the osmolality values in the silicate-based WE-PEO1 (0.49 ± 0.002 mOsm/Kg) group were significantly higher compared to the other coated materials, i.e., the WE43-PEO2 group up to the WE43-PEO11 group (*** *p* < 0.001) (Figure 3B). Also, the values in the WE43-PEO2 group (0.48 ± 0.002 mOsm/Kg), in the WE43-PEO3 group (0.43 ± 0.0006 mOsm/Kg) and in the WE43-PEO4 group (0.44 ± 0.003 mOsm/Kg) were significantly higher compared to the following study groups (*** *p* < 0.001) (Figure 4B). Thus, the osmolality values of the four silicate-based materials differed significantly compared to each other (Figure 3B).

Furthermore, the statistical comparison of the values in the phosphate-based Mg-material groups revealed that the values in the WE43-PEO5 group (0.40 ± 0.003 mOsm/Kg) differed significantly compared to the values in the WE43-PEO6 group (0.0.39 ± 0.001 mOsm/Kg, * *p* < 0.05), to the values in the WE43-PEO7 group (0.45 ± 0.005 mOsm/Kg, *** *p* < 0.001), to the values in the WE43-PEO8 group (0.38 ± 0.001 mOsm/Kg, *** *p* < 0.001), to the values in the WE43-PEO10 group (0.38 ± 0.002 mOsm/Kg, *** *p* < 0.001) and to the values in the WE43-PEO11 group (0.37 ± 0.001 mOsm/Kg, *** *p* < 0.001) but not compared to the WE43-PEO9 group (0.39 ± 0.002 mOsm/Kg) (Figure 3B). Additionally, the values in the WE43-PEO6 group differed significantly compared to the values in the WE43-PEO7 group, to the values in the WE43-PEO8 group (*** *p* < 0.001), to the values in the WE43-PEO10 group (*** *p* < 0.001) and to the values in the WE43-PEO11 group (*** *p* < 0.001) but also not compared to the WE43-PEO9 group (Figure 3B). The values in the WE43-PEO7 group were statistically higher compared to that in the groups of the WE43-PEO8 group to the WE43-PEO11 group (*** *p* < 0.001) (Figure 3B). The values in the WE43-8 group furthermore differed significantly compared to the values in the WE43-PEO9 group (*** *p* < 0.001) and to the value in the WE43-PEO11 group (*** *p* < 0.001) but not to that found in the WE43-PEO10 group (Figure 3B). The values in the WE-PEO9 group were also significantly higher to that found in the WE43-PEO10 group and the WE43-PEO11 group (*** *p* < 0.001) (Figure 3B). Moreover, the values in the WE43-PEO10 group were significantly higher compared to the values in the WE43-PEO11 group (*** *p* < 0.001) (Figure 3B).

Finally, all values in the groups of the Mg-based materials were significantly higher compared with the values in the control groups, i.e., the WAKO group, the RM-A group and both titanium groups that did not show any osmolality (*** *p* < 0.001) (Figure 3B).

#### 2.2.4. Cytocompatibility Analyses

**Figure 5:**
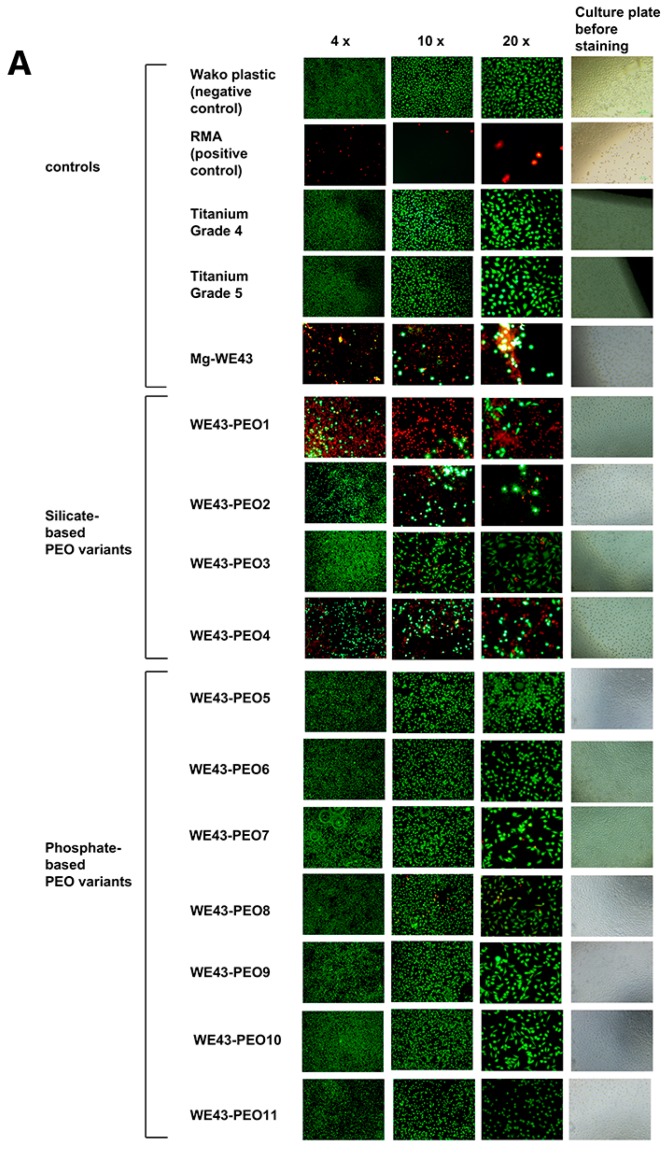

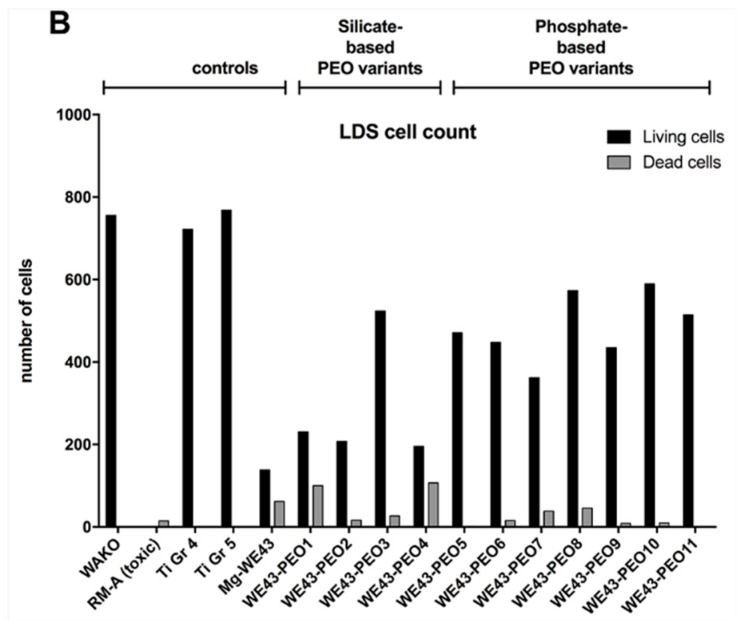
Attachment of cells on surfaces of the PEO variants. (**A**) Attachment, vitality and morphology of the cells. Green: vital cells; red: dead cells. Spindle shaped morphology indicates healthy cells with firm attachment. (**B**) Counted cells. Vital (green) and dead (red) cells were counted on the 100× magnification photos using the software ImageJ. Shown are mean and standard deviation from 3 experiments.

The measurements of the cytotoxicity assay showed that eight out of the eleven WE43-PEO-variants exhibited suitable cytocompatibility with values <130% of the negative control in the cytotoxicity assay and values >70% of the negative control in the viability and proliferation assays, which indicate the nontoxic range as defined in ISO 10993-5:2009 [23] (Figure 4. However, three silicate-based variants WE43-PEO1, WE43-PEO2 and WE43-PEO4 exhibited high cytotoxicity (Figure 4A), reduced viability (Figure 5B) and proliferation (Figure 4C). WE43-PEO1 and WE43-PEO2 showed values outside of the nontoxic range in all three assays while PEO4 exhibited viability >70% of the negative control in the viability assay but increased cytotoxicity (250% of NC) and decreased proliferation (62% of NC). WE43-PEO3 and WE43-PEO6 showed slightly increased values for cytotoxicity. Standard deviations were relatively low for variants with good cytocompatibility (not exceeding 30%) and higher for cytotoxic variants (up to 90%).

Unmodified Mg-WE43 is toxic and consequently did not enable adequate cell attachment (Figure 4A). Although some vital cells were visible, they exhibited a round morphology, suggesting lack of adhesion and spread of the cells on the surface. A similar morphology of the cells was also observed for several PEO-variants, for example for the silicate-based WE43-PEO2 and WE43-PEO4, suggesting unsatisfactory attachment. By contrast, on the surface of phosphate-based WE43-PEO5, WE43-PEO8, WE43-PEO9, and WE43-PEO10, natural cell morphology was visible, indicating satisfactory attachment of vital cells (Figure 5A).

Cell counting of the live-dead staining images revealed a tendency towards higher counts of living cells and lower counts of dead cells on the variants with phosphate-based surface-coatings (range 361–589 for living cells and 1–45 for dead cells) in comparison with the variants with silicate-based surface-coatings (range 139–523 for living cells and 16–107 for dead cells) (Figure 5B). An exception from this trend is the silicate-based PEO3 with a high count of 523 living cells, albeit with a high SD of 336. PEO8 and PEO10 exhibited the most living cells (573 and 589) while showing few dead cells (45 and 10) (Figure 5B).

Based on this first data set, four variants were selected for further characterization: PEO5, PEO8, PEO9, and PEO10.

### 2.3. Second Material Analyses

#### 2.3.1. Mechanical Analyses

**Figure 6:**
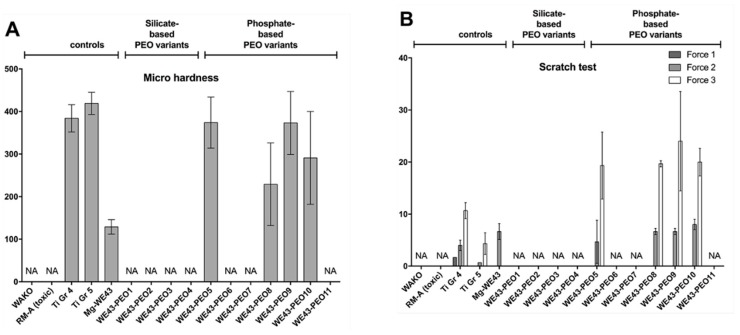
Mechanical features of PEO variants. (**A**) Stiffness; (**B**) Penetration. The left six specimens are controls and references. Column heights are means with error bars indicating standard deviations. NR: not relevant. NA: not analyzed.

Both titanium materials reached the highest values of micro hardness (Ti Gr. 4: 384 ± 32 HV 0.02 Ti Gr. 5: 419 ± 26 HV 0.02) as expected (Figure 6A). The stiffness of the base material, i.e., the Mg-alloy WE43, reached approximately 1/3 of that of titanium (129 ± 17 HV 0.02) (Figure 6A). After PEO surface treatment, WE43-PEO5 (374 ± 60 HV 0.02) and WE43-PEO9 (373 ± 74 HV 0.02) and showed values comparable to the values in both Ti groups in case of the variants WE43-PEO5 and -9 (Figure 6A). WE43-PEO8 (229 ± 97 HV 0.02) and WE43-PEO10 (291 ± 109 HV 0.02) revealed lower values between Mg-WE43 and titanium test samples (Figure 6A).

Moreover, the scratch tests using three different forces showed that only in case of the Ti Gr. 4 a force of 1.67 N was needed for first cracks, while all other materials did not resist this minimal force (Figure 6B). At LC_2_, comparable forces were measured in case of the Mg-alloy WE43 (6.67 ± 1.53 N) and the coating variants WE43-PEO8 (6.67 ± 0.58 N), WE43-PEO9 (6.67 ± 0.58 N) and WE43-PEO10 (8.00 ± 1.1 N), while lower forces were detected for the variant We43-PEO5 (4.67 ± 4.16 N) (Figure 6B). Also, a higher value was detected in the Ti Gr. 4 group (4.00 ± 1.00 N), while the lowest values were measured in the Ti Gr. 5 group (0.67 ± 0 N) (Figure 6B). At LC_3_ comparable high values were still found in the groups of the material variants WE43-PEO5 (19.33 ± 6.43 N), WE43-PEO8 (19.67 ± 0.58 N), WE43-PEO9 (24.0 ± 9.54 N), and WE43-PEO10 (20.0 ± 2.65 N) (Figure 6B). Again, lower values were measured in the Ti Gr. 4 group (10.67 ± 1.53 N), while the lowest values were detected in the Ti Gr. 5 group (4.33 ± 2.08 N) (Figure 6B).

#### 2.3.2. Analysis of the Osteogenic Differentiation of HUCPV Cells

**Figure 7:**
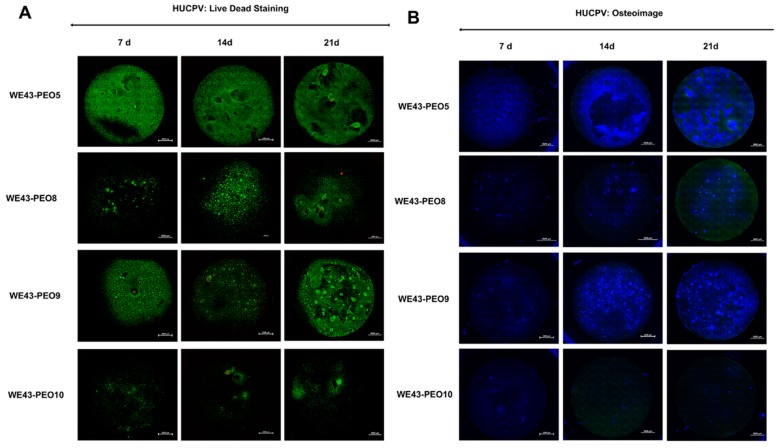
Attachment (**A**) and osteogenic differentiation (**B**) of Human umbilical cord perivascular cells (HUCPV) on the PEO variants. Mineralized bone-like nodules were visualized at days 7, 14, and 21 by staining a bone component hydroxyapatite (blue) using an OsteoImage kit (Lonza).

The co-cultivation of the selected material variants with human umbilical cord perivascular (HUCPV) cells revealed that vital cells already formed a consistent layer after 7 days on the surfaces of WE43-PEO5 and WE43-PEO9 without high numbers of dead cells at this early time point (Figure 7A). In the case of the variants WE43-PEO8 and WE43-PEO10, only loosely detectable vital cells were found but also without high numbers of dead cells (Figure 7A). Interestingly, this analysis step additionally showed that first signs of bone matrix deposition were seen in the WE43-PEO5, WE43-PEO8 and WE43-PEO9 groups (Figure 7A,B). After 14 days, a complete layer of vital cells was only found at the surfaces of the variant WE43-PEO5 without high numbers of dead cells, while moderate numbers of vital cells were found at the surfaces of the material variants WE43-PEO8 and WE43-PEO9 (Figure 7A). In case of the WE43-8 group, only single dead cells were detected, while in the WE43-PEO9 group higher numbers of dead cells were found (Figure 7A). In contrast, a low number of vital cells was found in the WE43-PEO10 group combined with moderate numbers of dead cells (Figure 7A). At this time point, comprehensive matrix deposition was only found in the WE43-PEO5 group, while also single calcification spots were found in the WE43-PEO9 group (Figure 7B). Moreover, only minor spots were found in the WE43-PEO8 group, while no calcification was seen in the WE43-PEO10 group (Figure 7B).

After 21 days, comprehensive layers of vital cells were observable combined with only single dead cells at the surfaces of the material variants WE43-PEO5 and WE43-PEO9 (Figure 7A). In the WE43-PEO8, moderate numbers of vital cells and single numbers of dead cells were found, while only single spots of vital cells were detected on WE43-PEO10 associated with lower numbers of dead cells (Figure 7B). Again, bone matrix associated with trabecula formation was found on the surfaces of the material variant WE43-PEO5, while higher numbers of bone nodule formation have been observed at the surfaces of the Variant WE43-PEO9 (Figure 7B). In the WE43-PEO8 group, single bone nodules were detectable, while in the WE43-PEO10 group show nearly no bone formation (Figure 7B).

## 3. Discussion

In the context of regenerative materials, biocompatibility can be defined as the harmless usability of a medical device for human use. Thereby, any potentially harmful physiological effects and major interferences on the harmonious biological functions should be excluded. Possible adverse effects can range from acute to chronic inflammation after implantation that can finally lead to implant failure [9,10,11]. Thus, medical devices have to undergo rigorous biological evaluation that should already begin very early in the development process to ensure successful interaction with the host. The ultimate aim is to protect patients from potential biological threats.

Especially the development of degradable biomaterials includes the evaluation of the degradation mechanisms to prevent any adverse effects from the liberated products. In this context, the ISO standards are of enormous importance for market approval of a medical device (CE certification). Any biomaterial must pass this test series, in which the test samples are tested in direct and indirect contact with mammalian cells (mainly used: L929 mouse fibroblasts). Only materials with sufficient cytocompatibility, as shown in the results section, pass the test procedure. All assays are relatively inexpensive, relatively fast to carry out and very reliable in their informative value. The resulting data allow a simple and standardized comparison with other biomaterials [12].

**Figure 8:**
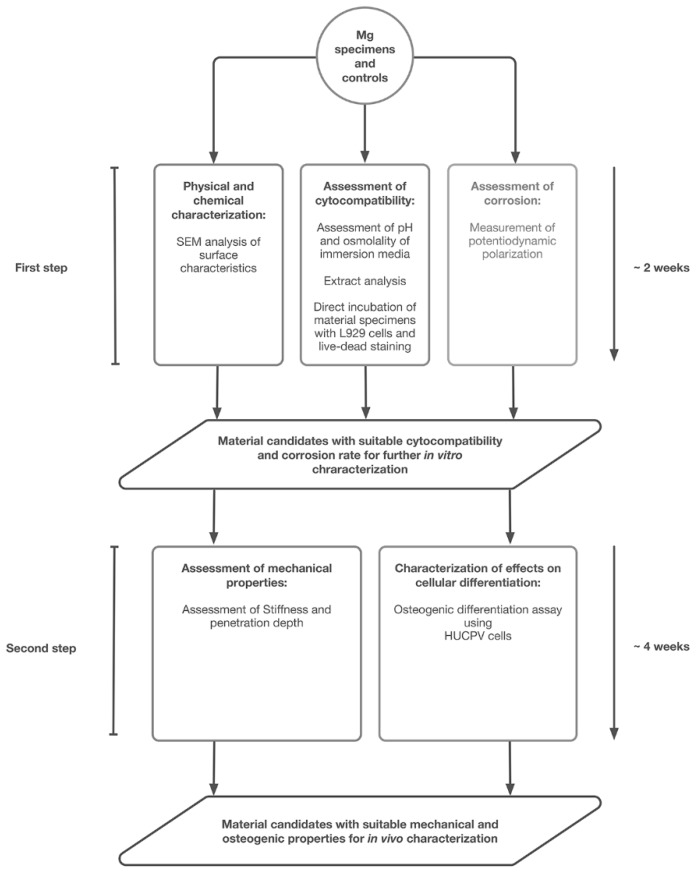
Schematic outline of the procedure for assessing the in vitro compatibility of Mg-based materials applied in this study.

Interestingly, this test series is used for the cytocompatibility testing of all classes of biomaterials. However, no official adaptions for new classes of biomaterials such as biodegradable biomaterials like magnesium have been developed until now [36,37]. In this context, Mg corrosion and thus its in vitro cytotoxicity has already shown to be influenced by various conditions and media-components such as pH and concentrations of HCO_3_^−^, albumin, etc. [26,27,28,30,31,37,38,39]. Moreover, magnesium-corrosion has already been reported to interfere with the commonly used assays [31]. Thus, an optimized and adjusted procedure for assessing the cytocompatibility of Mg-based biomaterials in concordance with ISO 10993-5/-12 has been reported in a previous study of our group [23]. However, this test protocol only includes cytocompatibility analyses for this new material class, while important aspects such as quantitative magnesium degradation, mechanics or regenerative bone capacities are disregarded. The present study was conducted to extend the solitary cytocompatibility procedure to include mechanical degradation and osteogenic properties. The newly developed test series represents a simple und time-saving step-wise approach to allow a safe selection of biomaterials for further in vivo analysis (Figure 8). The approach was established by testing a series of 11 Mg-based biomaterials with different surface modifications by means of plasma electrolytic oxidation (PEO).

Initially, eleven test samples were manufactured using different electrolytical compositions. SEM showed that the ceramized test samples showed various degrees of surface roughness and microporosity in comparison to the untreated Mg alloy WE43. Corrosion measurements revealed promising results for all PEO test samples except from WE43-PEO7, WE43-PEO8, and WE43-PEO11 (Table 1). pH and osmolality represent important corrosion markers since Mg degradation increases both of the attributes. Thereby, favorable pH and osmolality could be detected for WE43-PEO5, WE43-PEO6, and WE43-PEO8-11 (Table 1).

The last analysis section of the first step revealed favorable cytocompatibility, pH, and osmolality for almost all phosphate-based test samples, while the silicate-based test samples must be classified as toxic (Table 1). WE43-PEO7, WE43-PEO8 and WE43-PEO11 revealed high corrosion currents, which is partially substantiated by high pH and osmolality values for WE43-PEO7. WE43-PEO6 showed increased cytotoxicity values, which makes it unsuitable for any in vivo-deployment. Additionally, WE43-PEO11 exhibited a rounded cell morphology on the test samples as an expression of low cell adherence. Overall, WE43-PEO5, WE-43-PEO9, and WE43-10 could fulfill all the criteria of the first step, while WE43-PEO6 showed increased cytotoxicity and WE-43-PEO8 higher corrosion values. However, we assessed increased cytotoxicity values as incompatible for any in vivo-experiments, while the higher degradation kinetics can be attributed to methodical interferences since pH and osmolality were suitable for WE43-PEO0 [40].

In this context, it has manifoldly been described that different physical and chemical properties of a biomaterial can have major influence on cytocompatibility [4,12,13]. In the present study, only a correlation between pH, osmolality, and cytocompatibility measurements could be shown. In a previous study of our group, we could demonstrate that grooved and microporous PEO surface patterns, comparable with WE43-PEO5 to-10, can lead to favorable cell adherence [20]. However, no straightforward correlation was observed between corrosion currents of the PEO variants and their chemical properties as well as their cytocompatibility. In this context, it is assumable that PDP can serve as a fast method to detect magnesium corrosion, but should not be overrated due to some inaccuracies [40]. Thus, the related release of hydrogen that is linked to the pH and the osmolality seemed to be the more accurate aspect in case of the analyzed material variants. Moreover, on basis of the present results, it becomes very clear that the phosphate-base coating variants outperform the silicate-based coating variants. In this context, it has already been described by Ma et al. that ceramization with phosphate-based electrolytes allowed a better corrosion resistance of magnesium than those produced from silicate [19]. Altogether, the results of this initial biomaterial testing series showed that only four phosphate-ceramized materials, WE43-PEO5, WE43-PEO8, WE43-PEO9, and WE43-PEO10, could be selected from the total number of PEO variants based on their superior properties as eligible materials for implants. Thus, these material variants were used for further analysis in a second step.

The results in the second testing step showed favorable micro-hardness values for WE43-PEO5 and WE43-PEO9 that were comparable to that of the tested titanium materials. Also, in case of the coating variants WE43-PEO10 and WE43-PEO8, higher values in comparison the pure Mg alloy WE43 were found but they were clearly decreased compared to the values in both titanium groups. Moreover, the scratch test results revealed only minor differences between the four coating variants. However, on basis of the co-cultivation of the selected material variants with human umbilical cord perivascular (HUCPV) cells it became very clear that only the Material variants WE43-PEO5 and -9 allowed for an undisturbed cell growth and, moreover, for a cell differentiation pattern that leads to bone matrix deposition in contrast to the two other variants.

Taken together, these WE43-PEO5 and WE43-PEO9 could be identified out of eleven tested materials based on the combination of conventional biocompatibility testing methods in combination with a new testing step including analyses of the of the mechanical and osteo-stimulative material characteristics. As a result, these two variants were identified as promising candidates for further in vivo characterization. Altogether, the results of this study show that the presented newly developed step-wise approach (Figure 8) enables for a rapid and effective identification of Mg-based biomaterial candidates in vitro and might excellently be suitable for prevention of unnecessary in vivo testing.

## 4. Materials and Methods

### 4.1. Biomaterial Preparation and Characterization

#### 4.1.1. Plasma Electrolytic Oxidation (PEO) Treatment of Magnesium Specimens

Disks made of magnesium alloy WE43 were cut from a rod with the dimensions 20.6 mm diameter and 1.5 mm thickness. They were subsequently cut-grinded with a silicon carbide (SiC)-wheel, etched in oxalic acid and rinsed in deionized water before subjecting to surface modification by means of plasma electrolytic oxidation (PEO) in a cool jacketed steel container serving as the cathode. The PEO treatment was carried out using a pulsed rectifier set (M-PEO A1, Meotec, Boulogne-Billancourt, France) at 10 Hz for 15–45 min under currents between 1.6 and 4.9 A with according voltages up to 500 V and in various electrolytes (Table 2). Individual parameter sets were adjusted specifically to each electrolyte in order to achieve a common coating thickness of 10 ± 3.5 μm. The facilitated electrolytes could be divided into two groups according to their major components: silicate-based and phosphate-based coatings. A total of four silicate-based and seven phosphate-based PEO-modifications with various surface roughnesses and structures were prepared: WE43-PEO1 to -4 (silicate group) and WE43-PEO5 to -11 (phosphate group).

#### 4.1.2. Physicochemical Material Characterization

The physical and chemical compositions of the test samples were analyzed scanning electron microscopy (SEM) (XL30 CP SEM, Philips, Amsterdam, The Netherlands) and energy dispersive X-ray spectroscopy (EDX).

#### 4.1.3. Assessment of Corrosion by Potentiodynamic Polarization Measurements (PDP)

Corrosion of all test samples was measured using potentiodynamic polarization measurements (PDP). Testing was performed in a three-electrode cell. The experimental setup included a potentiostat (SP-150 153, Bio-Logic Science Instruments, Seyssinet-Pariset, France), a glass corrosion cell equipped with minimum essential medium (MEM, Life Technologies, Carlsbad, Germany), an Ag/AgCl, NaCl saturated electrode and a graphite rod as the counter electrode. Cell culture medium was chosen for ease of use due to its comparable chloride. In order to achieve a more reliable electrical contact of the working electrode (specimen) and improved conductive circuit, the PEO surface was locally removed from the site of contact. Degradation material was kept in a controlled environment of 37 °C and 5% CO_2_. Open circuit potential (OCP) variations were recorded continuously during the immersion time of 1 h. Polarization curves were registered at a scan rate of 30 mV/min within a range of −500 mV to +500 mV, respectively to the OCP. Corresponding measurements were calculated according to Ascencio et al. [40,41].

#### 4.1.4. Determination of pH and Osmolality of Immersion Medium

pH and osmolality after Mg corrosion were measured after keeping the test samples in MEM (Minimum Essential Medium) supplemented with 10% fetal bovine serum, penicillin/streptomycin (100 U/mL each) (all from Life Technologies, Carlsbad, CA, USA) and L-glutamine (Sigma-Aldrich, St. Louis, MO, USA) to a final concentration of 4 mM (in the following referred to as cell culture medium) at 37 °C, 5% CO_2_, and 95% humidity (cell culture conditions) for 72 h. A surface to volume ratio of 3 cm^2^/mL was maintained.

### 4.2. Cytocompatibility Analyses

Cytocompatibility analyses were conducted in accordance with the ISO 10993-5/-12 and were already described in detail previously [12]. The experimental setup is described briefly in the following paragraphs.

#### 4.2.1. Reference Materials (Positive and Negative Controls)

All samples were sterilized by immersion in isopropanol for 5 min with subsequent drying in a laminar flow hood. RM-A, a polyurethane film containing 0.1% zinc diethyldithiocarbamate (ZDEC) that was obtained from the Hatano Research Institute, Food and Drug Safety Center, Japan was used as a positive control reference material. For live-dead staining assays, Wako plastic sheets (Wako Pure Chemical Industries, Ltd., Osaka, Japan, Cat. No.160-08893) were used as a nontoxic control material. Additionally, sterilized pure grade 4 and 5 titanium (Ti) materials were utilized as controls. Samples of RM-A, Wako plastic sheets and titanium were prepared with the same surface areas as the material specimens and sterilized likewise.

#### 4.2.2. Cell Culture

L-929 mouse fibroblasts were obtained from the European Collection of Cell Culture, ECACC (Salisbury, UK). Cells were cultured in cell culture medium under standard cell cultured conditions. At about 80% confluency, cells were passaged.

#### 4.2.3. Extract Analysis

##### Extraction

Test- and control samples were extracted for 72 h at a surface to volume ratio of 3 cm^2^/mL in cell culture medium under cell culture conditions. Cell culture medium alone was incubated under identical conditions to serve as a negative control extract. After removal of the specimens, the remaining extracts were centrifuged at 14,000 rpm for 10 min. The supernatants were used for the different assays that described below.

##### Assay Procedure

96 well plates were seeded with 1 × 10^4^ L929 cells/well in 100 μL cell culture medium and incubated under cell culture conditions for 24 h. Thereafter, cell culture medium was discarded and 100 μL of extract were added to each well. Cells were further incubated for 24 h and then subjected to the BrdU- and XTT-assays while the supernatants were subjected to the Lactate Dehydrogenase (LDH)-assay. Identical assays but omitting cells were conducted for all extracts as a control for assay interference. Blank controls (medium alone without cells) were subtracted from the absorbance values in all assays.

##### Bromodeoxyuridine/5-Bromo-2′-Deoxyuridine (BrdU)-Assay

BrdU (colorimetric) test kit (Roche Diagnostics, Mannheim, Germany) was used according to the manufacturer’s instructions. Briefly, cells were labeled with BrdU for 2 h under cell culture conditions and subsequently fixed for 30 min at room temperature with FixDenat reagent. Then, the fixed cells were incubated for 1 h with anti-BrdU-peroxidase (POD) antibody and washed 3 times for 5 min with washing buffer. The immune complexes were detected after a subsequent substrate reaction with tetramethyl-benzidine (TMB) (20 min at room temperature) followed by addition of 25 μL 1 M H_2_SO_4_ to stop the reaction using a scanning multi-well spectrophotometer (ELISA reader) with filters for 450 and 690 nm (reference wavelength).

##### Sodium 3,3′-[1(Phenylamino)Carbonyl]-3,4-Tetrazolium]-3is(4-Methoxy-6-Nitro) Benzene Sulfonic acid Hydrate (XTT)-Assay

The Cell Proliferation Kit II (Roche Diagnostics, Mannheim, Germany) was used according to the manufacturer’s instructions. Briefly, the electron-coupling reagent was mixed with XTT labeling reagent (1:50 dilution) and 50 μL of the mixture was added to the cells. After 4 h of incubation under cell culture conditions, substrate conversion was quantified by measuring the absorbance of 100 μL aliquots in a new 96 well plate using a scanning multi-well spectrophotometer (ELISA reader) with filters for 450 and 650 nm (reference wavelength).

##### Lactate Dehydrogenase (LDH)-Assay

The LDH-Cytotoxicity Assay Kit II (BioVision, Milpitas, CA, USA) was used according to the manufacturer’s instructions. Briefly, 10 μL of the cell supernatants were incubated with 100 μL LDH reaction mix for 30 min at room temperature. After addition of stopping solution, absorbances were measured using a scanning multi-well spectrophotometer (ELISA reader) with filters for 450 and 650 nm (reference wavelength).

#### 4.2.4. Live-Dead Staining

Mg specimens and controls were seeded with 2.4 × 10^5^ cells in 1 mL medium in each well of 12 well plates (the surface-area/medium ratio was 5.65 cm^2^/mL). Assays were carried out after 24 h incubation under cell culture conditions. In order to perform live-dead cell staining on the surfaces of the specimens, 60 μL per ml medium propidium iodide (PI) stock solution (50 μg/mL in PBS) and 500 μL per ml medium fresh fluorescein diacetate (FDA) working solution (20 μg/mL in PBS from 5 mg/mL FDA in acetone stock solution) were added to each well (12 well plate). After a brief incubation for 3 min at room temperature, specimens were rinsed in prewarmed PBS and were immediately examined with an upright fluorescence microscope (Nikon ECLIPSE Ti-S/L100, Nikon GmbH, Düsseldorf, Germany) equipped with a filter for parallel detection of red and green fluorescence. Pictures were taken using a 4×, 10× and 20× objective. Cells visualized using the 10× objective were counted using the software ImageJ [12]. For each material, cells were counted from three experiments to calculate mean and standard deviation.

### 4.3. Analyses of the Bone Regeneration Characteristics

#### 4.3.1. Mechanical Characterization

Micro-hardness of the materials and evaluation of the layer quality were measured for four variants from the phosphate-group based on the results described above. For mechanical characterization of the PEO coatings as well as of the adhesion strength between coatings and substrates ultra-micro-hardness tests and scratch tests were performed. Within the ultra-micro hardness measurements Vickers hardness HV 0.02 was determined using a Dynamic Ultra Micro Hardness Tester (Shimadzu DUH-211, Kyoto, Japan). Scratch tests were performed using a CSM (Neuchâtel, Switzerland). The load on the Rockwell indenter was linearly increased from 0 N to a maximum load of 100 N over a scratch trace length of 10 mm. After the tests, critical loads LC_1_ to LC_3_ were determined by examination of the scratch trace using a scanning electron microscope, where LC_1_ indicates the critical load leading to first cracks in the coating. At LC_2_ first spallings occur at the edge of the scratch trace. LC_3_ is characterized by discontinuous, ductile perforation of the coating.

#### 4.3.2. Osteogenic Differentiation Assay

HUCPV were isolated with the approval from the local ethical committee “Ethik-Kommission der Ärztekammer Hamburg” (Hamburg, Germany). The written consent of the donor was obtained. The isolation and preparation of the cells was achieved as shown by Cecchinato et al. [30]. After cutting the umbilical cord into 5 cm pieces, they were cultured for 10 days in T-175 cell culture flask with α-MEM, 15% FBS and 1% antibiotics. When cells were outgrowing, the medium was changed every 2–3 days. When cells recached 80% confluency, they were detached and transferred into fresh medium.

100,000 human umbilical cord perivascular cells (HUCPV) were seeded onto four WE43-PEO variants with phosphate-based surface-coating under cell culture conditions: PEO5, PEO8, PEO9 and PEO10, and allowed to attach overnight. On the next day, the medium was changed to osteogenesis medium. After 3 weeks of osteogenic differentiation, the WE43 variants were stained with live-dead markers (Invitrogen, Carlsbad, CA, USA, catalogue: L-3224) and an OsteoImage kit (Lonza, Walkersville, MD, USA, catalogue: PA-1503) according the instructions of the manufacturer. The images were visualized using a fluorescence microscope (Nikon ECLIPSE Ti-S/L100, Nikon GmbH, Düsseldorf, Germany) and qualitatively analyzed.

### 4.4. Statistical Analyses

The data won by the different afore-mentioned analysis methods were statistically analyzed by an analysis of variance (ANOVA) combined with a Tukey’s multiple comparisons test via the GraphPad Prism 7.0d software (GraphPad Software Inc., San Diego, CA, USA). Statistical differences were designated as significant if p-values were less than 0.05 (* *p* ≤ 0.05), and highly significant if *p*-values were less than 0.01 (** *p* ≤ 0.01) or less than 0.001 (*** *p* ≤ 0.001).

## 5. Conclusions

The newly developed analysis protocol presented in this study includes a step-wise approach which allows a rapid and effective in vitro compatibility analysis of biodegradable magnesium alloys. Thereby, all aspects regarding corrosion, cytocompatibility, mechanics, and osteogenic properties could be considered.

We successfully demonstrated our test scheme by using 11 differently PEO-ceramized magnesium test samples in comparison with an untreated WE43 test samples. Essential but less elaborate and time-consuming assays were carried out in a first step while more sophisticated and time-intensive experiments were carried out with material variants that passed the first test series. Overall, two variants were able to convince in almost all aspects and can be potentially be employed for further in vivo-studies.

## Figures and Tables

**Table 1:** Evaluation scheme of each magnesium test sample. X indicates favorable results for the individual test sample. After each step, X were summed to obtain an overall score. Cor: Corrosion current, Osm: Osmolality, Cytotox: cytotoxcicity, Prolifer: proliferation, LDS Ql/Qn: Live-dead cell count qualitative/quantitative, Micro: micro hardness, Scratch: scratch test, Oseto: osteogenic properties.

	Cor	pH	Osm	Cytotox	Viability	Prolifer	LDS Ql.	LDS Qn.	1st Step	Micro	Scratch	Osteo	2nd Step
Mg-WE43									0				0
WE43-PEO1	X								1				
WE43-PEO2	X								1				
WE43-PEO3	X				X	X		X	3				
WE43-PEO4	X				X				1				
WE43-PEO5	X	X	X	X	X	X	X	X	**8**	X		X	**2**
WE43-PEO6	X	X	X		X	X	X	X	7				
WE43-PEO7				X	X	X	X	X	5				
WE43-PEO8		X	X	X	X	X	X	X	**7**		X		1
WE43-PEO9	X	X	X	X	X	X	X	X	**8**	X		X	**2**
WE43-PEO10	X	X	X	X	X	X	X	X	**8**		X		1
WE43-PEO11		X	X	X	X	X		X	6				

**Table 2:** Chemical composition of the electrolyte blends used to coat the surface of the Mg alloy WE43.

Variant	Electrolytes
Untreated	Magnesium WE43
Silicate-based	
PEO1	Silicate + Potassium Hydroxide
PEO2	Silicate + Potassium Hydroxide + Borate
PEO3	Silicate + Potassium Hydroxide + Titanate
PEO4	Silicate + Potassium Hydroxide + Borate + Titanate
Phosphate-based	
PEO5	Phosphate + Potassium Hydroxide
PEO6	Phosphate + Ammonium Hydroxide
PEO7	Phosphate + Potassium Hydroxide + Aluminate
PEO8	Phosphate + Ammonium Hydroxide + Urea
PEO9	Phosphate + Ammonium Hydroxide + EDTA
PEO10	Phosphate + Ammonium Hydroxide + Flouride + Urotropin
PEO11	Phosphate + Ammonium Hydroxide + Fluoride + Borate + Urotropin
